# Supplementary material for: A universal standardized method for output capability assessment of nanogenerators
Source: Nat Commun. 2019 Sep 27;10:4428. doi: 10.1038/s41467-019-12465-2 (PMC6765008; doi:10.1038/s41467-019-12465-2)
Supplement: Supplementary file 1 — Supplementary Information [file 41467_2019_12465_MOESM1_ESM.pdf]

Supplementary information

A universal standardized method for output capability assessment of nanogenerators

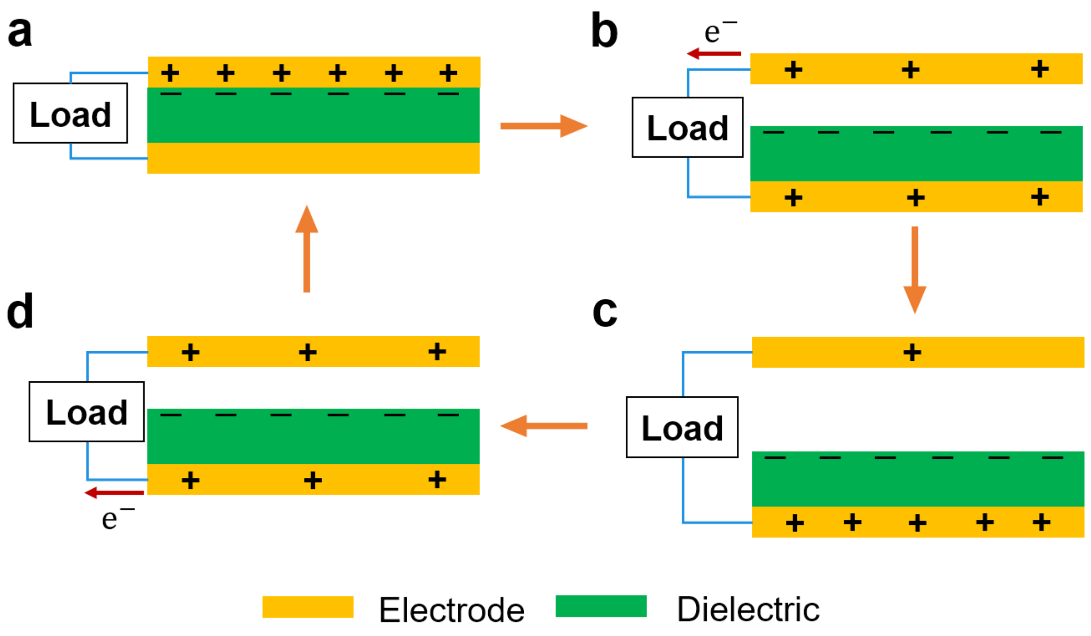

Supplementary Figure 1| Operation principle of CS mode TENG. (**a**) Initial charges of TENG is generated when the tribo-layers contact each other. (**b**) When the top layer starts to move away, electrons will transfer from top electrode to bottom electrode. (**c**) When separation displacement reaches x_{max}, the top layer starts to move back. (**d**) Electrons transfer from bottom electrode back to top electrode.

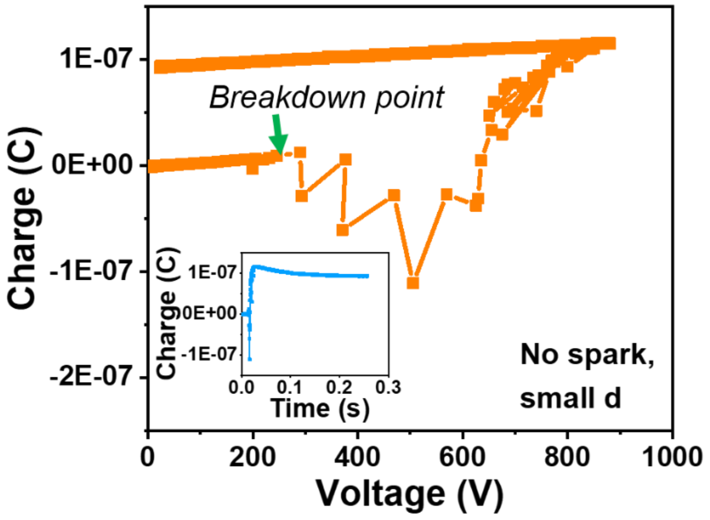

Supplementary Figure 2| Output characteristic of breakdown for CS mode with small displacement. When displacement is small, CS mode TENG is more prone to

breakdown and the $Q(x)$-$V(x)$ plot is more disordered. The breakdown point should be the point targeted by the green arrow after which the first peak shows.

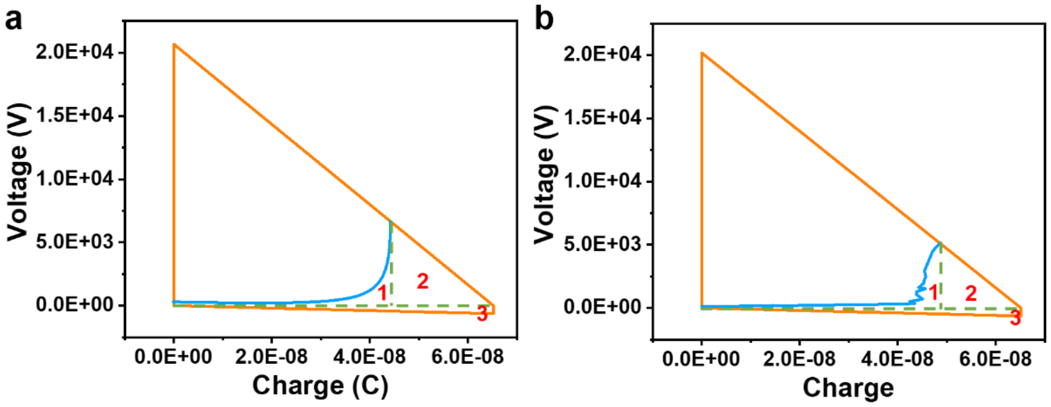

Supplementary Figure 3| Cycle of maximized energy output of CS mode TENG. *V*-*Q* plot with (**a**) theoretical maximized effective energy area and (**b**) measured maximized effective energy (E_{em}) area of CS mode TENG. Calculating parts are marked with numbers.

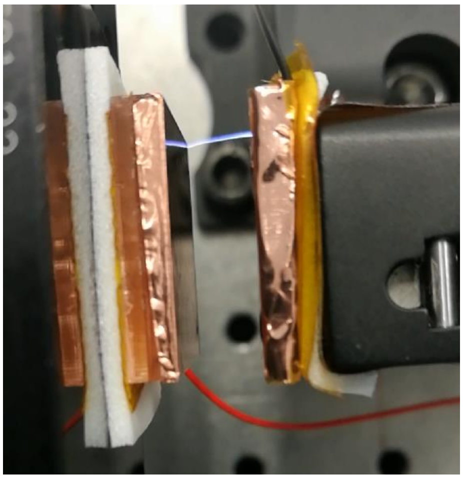

Supplementary Figure 4| Photo of spark when x = 5mm. This photo demonstrates that the proposed method is applicable for larger x_{max}.

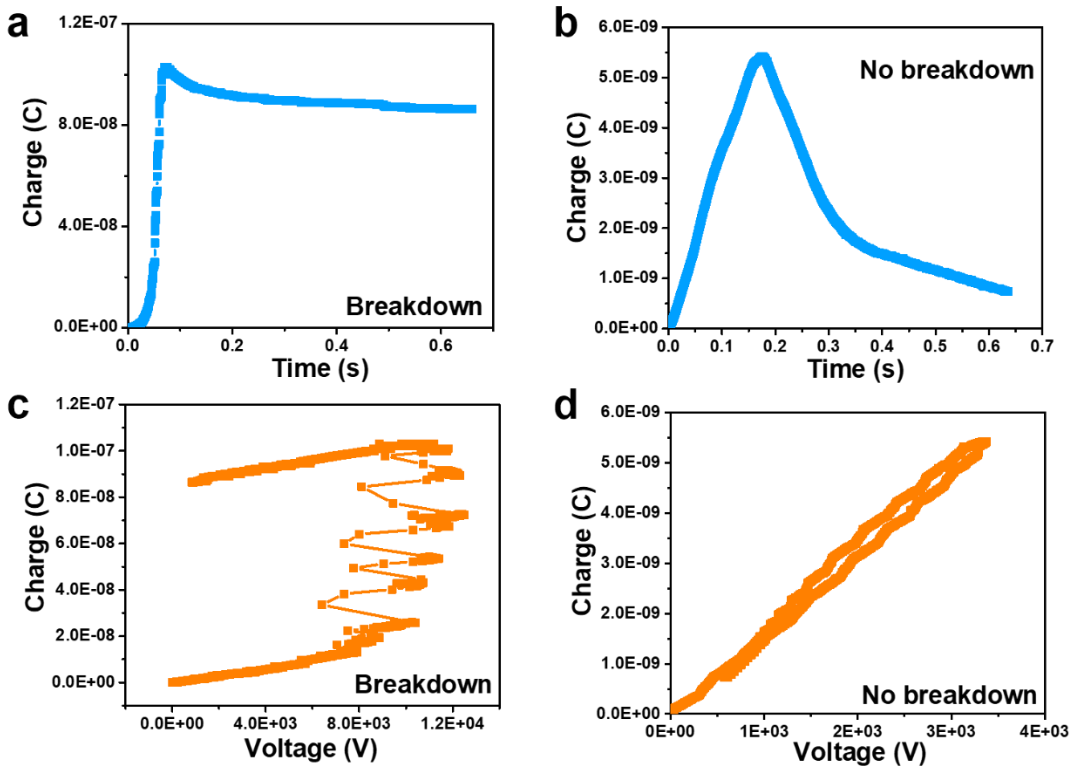

Supplementary Figure 5| Output characteristic of breakdown for CFT mode TENG. Transferred charge characteristic for CFT mode (**a**) with breakdown; (**b**)without breakdown. *Q*-*V* plots of CFT mode, (**c**) with breakdown; (**d**) without breakdown.

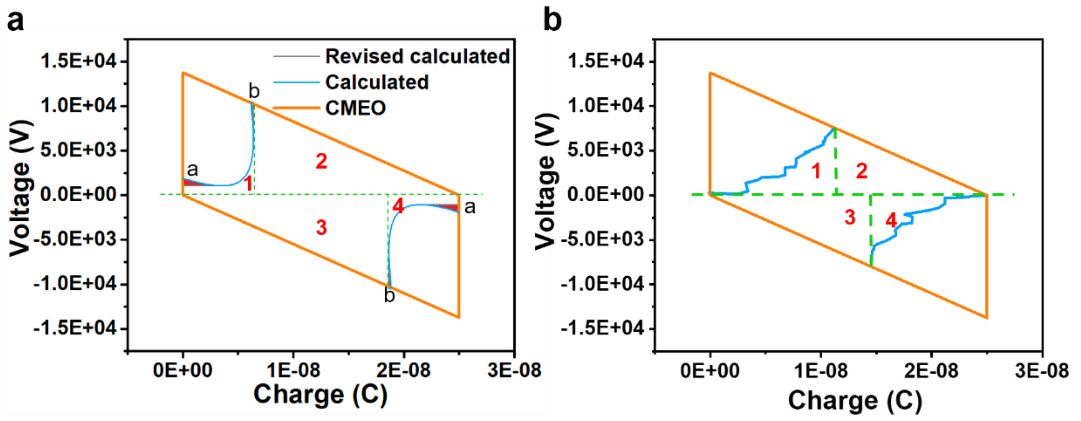

Supplementary Figure 6| Cycle of maximized energy output of CS mode TENG. (**a**) CMEO curve with theoretical E_{em}, plotted in blue and revised E_{em}, plotted in gray of CFT mode TENG. (**b**) CMEO curve with measured E_{em} of CFT mode TENG. Calculating parts are marked with numbers.

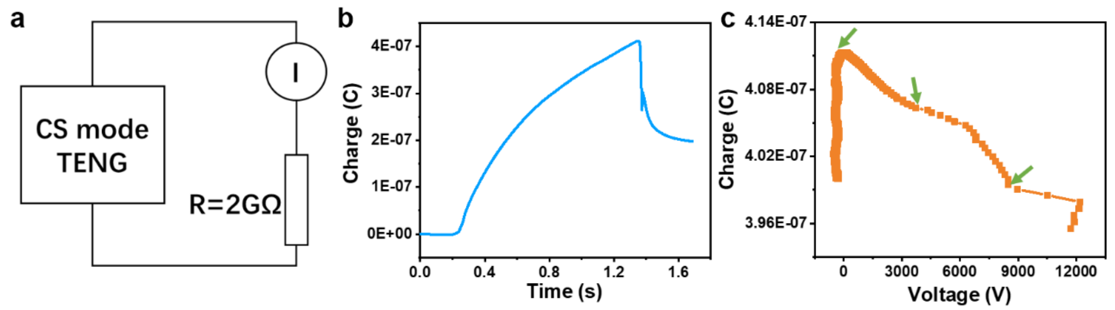

Supplementary Figure 7| Real-time measurement with the varied distance. (**a**) Experimental circuit for real-time measurement results. (**b**) Real-time *Q*-*t* plot. (**c**) Real-time *Q*-*V* plot. Points targeted by green arrows in(**c**) are the suspected breakdown points. Surface charge density of the CS mode TENG in this experiment is $120\mu\mathrm{C\ m^{-2}}$.

Supplementary Note 1. Calculation of E_{em} and FOM of CS mode TENG. The maximized effective energy E_{em} can be derived by calculating the area that no breakdown happens. In Supplementary Figure 3, the E_{em} is divided into 3 parts. Calculation results are list below:

Maximized energy output per cycle:

E_m = 695.012 μJ

Calculated E_{em}:

Area1 = 23.21512 μJ

Area2 = 69.97127 μJ

Area3 = 20.668 μJ

E_{em}= Area1 + Area2 + Area3 = 113.8544 μJ

Measured E_{em}:

Area1 = 25.77959 μJ

Area2 = 52.7401 μJ

Area3 = 20.668 μJ

E_{em} = Area1 + Area2 + Area3 = 99.1877 μJ

$$\mathrm{FOM_S} = \frac{2\varepsilon_0}{\sigma^2}\frac{E_{em}}{Ax_{max}} = \frac{2\times 8.85\times 10^{-12}}{(168\times 10^{-6})^2}\times\frac{99.1877\times 10^{-6}}{0.02\times 0.02\times 0.002} = 0.077754$$

$$\mathrm{FOM_M} = \sigma^2 = (168\times 10^{-3}\mathrm{mC\ m^{-2}})^2 = 0.028224(\mathrm{mC\ m^{-2}})^2$$

$$\mathrm{FOM_P} = 2\varepsilon_0\frac{E_{em}}{Ax_{max}} = 0.077754\times 0.028224 = 2.19453\times 10^{-3}(\mathrm{mC\ m^{-2}})^2$$

Supplementary Note 2. Calculation of E_{em} and FOM of CFT mode TENG. In Supplementary Figure 6, the E_{em} is divided into 4 parts. Total effective energy can be derived by integrating each part individually and then calculating the sum of each part. Calculation process is similar to CS mode, but the calculated E_{em} of CFT mode TENG should be revised, since it is not practical for any TENG turning to a low charge and

high voltage condition from a high charge and low voltage condition to avoid air breakdown (red area in Supplementary Figure 7(a)). Thus the calculated E_{em} was revised by making the larger voltage part (area (a) in red) equal to the lowest voltage and the larger charge part (area (b) in red) equal to the lowest charge.

Maximized energy output per cycle:
E_m = 343.68 μJ
Theoretical E_{em}:
Area1 = 8.9015 μJ
Area2 = 97.8296 μJ
Area3 = 98.8295 μJ
Area4 = 8.93804 μJ
E_{em} = Area1 + Area2 + Area3 + Area4 = 214.4986 μJ
Measured E_{em}:
Area1 = 29.78 μJ
Area2 = 52.08 μJ
Area3 = 57.80 μJ
Area4 = 22.04 μJ
E_{em} = Area1 + Area2 + Area3 + Area4 = 161.7 μJ

$$\mathrm{FOM_S} = \frac{2\varepsilon_0}{\sigma^2}\frac{E_{em}}{Ax_{max}} = \frac{2\times 8.85\times 10^{-12}}{(38.6\times 10^{-6})^2}\times\frac{167\times 10^{-6}}{0.02\times 0.02\times 0.002} = 2.40115$$

$$\mathrm{FOM_M} = \sigma^2 = (38.6\times 10^{-3}\mathrm{mC\ m^{-2}})^2 = 1.48996\times 10^{-3}(\mathrm{mC\ m^{-2}})^2$$

$$\mathrm{FOM_P} = 2\varepsilon_0\frac{E_{em}}{Ax_{max}} = 2.40115\times 1.48996\times 10^{-3} = 3.5776\times 10^{-3}(\mathrm{mC\ m^{-2}})^2$$

Supplementary Note 3. Calculation of E_{em} and FOM of PVDF film-based PENG. Simply applying the revised FOM equation on PVDF film-based PENG, thus the measured FOM are calculated as follows:

$$E_{em} = Q_{SC,max}\times V_{OC,max} = 54.3\times 10^{-9}\times 134 = 7.2762\times 10^{-6}\mathrm{J} = 7.2762\mu\mathrm{J}$$

$$\sigma = \frac{Q_{SC,max}}{A} = \frac{54.3\times 10^{-6}}{0.01\times 0.012} = 452.5\times 10^{-6}\mu\mathrm{C\ m^{-2}}$$

$$\mathrm{FOM_S} = \frac{2\varepsilon_0}{\sigma^2}\frac{E_{em}}{Ax_{max}} = \frac{2\times 8.85\times 10^{-12}}{(452.5\times 10^{-6})^2}\times\frac{7.2762\times 10^{-10}}{0.01\times 0.012\times 0.02} = 2.62077\times 10^{-4}$$

$$\mathrm{FOM_M} = \sigma^2 = (452.5\times 10^{-3}\mathrm{mC\ m^{-2}})^2 = 0.204756(\mathrm{mC\ m^{-2}})^2$$

$$\mathrm{FOM_P} = 2\varepsilon_0\frac{E_{em}}{Ax_{max}} = 2.62077\times 10^{-4}\times 0.204756 = 5.36620\times 10^{-5}(\mathrm{mC\ m^{-2}})^2$$

Supplementary Note 4. Real-time measurement results of CS mode TENG. The real-time *Q*-*V* plot can also be measured by directly connected the TENG with the external resistance, as shown in Supplementary Figure 8(a). *Q* and *V* are obtained based on the equations shown below:

$$Q = \Sigma i\Delta t$$

$$V = iR$$

The real-time Q-t plot is as shown in Supplementary Figure 8(b). Supplementary Figure 8(c) shows the real-time Q-V plot. Similar to the static measurement results, when breakdown happens, there are turning points observed with sparks, which can be considered as the breakdown points. However, in fact we can only mark a few suspected breakdown points by the green arrows in Supplementary Figure 8(c) since sparks can be only observed sometimes to confirm it. The video record of the real-time sparks is added in the Supplementary Video 5. Therefore, the reasons that we cannot use this real-time Q-V measurement method are as below: Firstly, these turning points are quite hard to be identified by the measured curves only, since the real-time curves in non-breakdown areas are usually not straight lines, as demonstrated in our previous work.[1] Secondly, this method cannot guarantee the measurement of breakdown points at all displacements since the displacement is keeping varying. Thirdly, we cannot extract the real capacitance of the TENG from the curves to validate our measurement due to the time-varied capacitance. Hence, the static Q-V plot based method is developed as a universal method in the manuscript.

Supplementary Video 1. Dynamic measurement for output characteristic of CS mode TENG.

Supplementary Video 2. Dynamic monitoring for air breakdown with visible sparks of CS mode TENG.

Supplementary Video 3. Dynamic monitoring for air breakdown with visible sparks of CFT mode TENG.

Supplementary Video 4. Dynamic monitoring for air breakdown with visible sparks of PVDF film-based PENG.

Supplementary Video 5. The real-time sparks recording of the CS mode TENG ($\mathbf{10cm \times 10cm}$).

Supplementary References

1. Zi, Y. *et al.* Standards and figure-of-merits for quantifying the performance of triboelectric nanogenerators. *Nat. Commun.* **6**, 8376, (2015).
